# Clinical relevance of molecular aspects in extranodal marginal zone lymphoma: a critical appraisal

**DOI:** 10.1177/17588359231183565

**Published:** 2023-06-22

**Authors:** Markus Raderer, Barbara Kiesewetter, Ming-Qing Du

**Affiliations:** Department of Medicine I, Division of Oncology, Medical University of Vienna, Waehringer Guertel 18 – 20, Vienna, A-1090, Austria; Department of Medicine I, Division of Oncology, Medical University of Vienna, Austria; Division of Cellular and Molecular Pathology, Department of Pathology, University of Cambridge, Cambridge, UK

**Keywords:** extranodal lymphoma, gastric lymphoma, *H. pylori*, MALT lymphoma, NF-kB

## Abstract

Extranodal marginal zone B-cell lymphoma of the mucosa-associated lymphoid tissue (MALT lymphoma) is among the more common types of lymphoma accounting for up to 8% of newly diagnosed lymphoma cases. As opposed to other B-cell lymphomas, however, no predominant genetic hallmark has been defined in MALT lymphoma, but different localizations appear to be affected by different, sometimes distinct changes. Nonetheless, a high proportion of these genetic changes reported in MALT lymphomas dysregulate the pathways leading to NF-kB activation. t(11;18)(q21;q21)/BIRC3::MALT1 appears to be MALT lymphoma specific and is found in 24% of gastric and 40% of pulmonary MALT lymphomas. The translocation is associated with more disseminated disease in gastric MALT lymphoma and is found in a large percentage of patients whose lymphoma is unresponsive to antibiotic eradication of *Helicobacter pylori.* In addition to t(11;18)(q21;q21), nuclear expression of BCL10 or NF-kB appears to be highly associated with lymphoma cell survival independence of *H. pylori-*mediated stimulations. Antibiotic eradication, however, is the recommended therapy of choice irrespective of genetic findings, and molecular analysis is not required before initiation of therapy. The influence of genetic translocations including t(11;18)(q21;q21) on systemic therapies, however, is less clearly defined. While small series have shown no influence on the outcome for treatment with the anti-CD20 antibody rituximab (R) or treatment with cladribine (2-CdA), conflicting data have been reported for alkylating agents, especially chlorambucil and the combination of *R* + chlorambucil. None of other genetic changes seen in MALT lymphoma to date has discernible value in routine clinical applications, but recent data suggest that changes in *TNFAIP3*(A20), *KMTD2* and *CARD11* might be associated with response to Bruton kinase inhibitors.

## Introduction

Extranodal marginal zone B-cell lymphoma of the mucosa -associated lymphoid tissue (MALT lymphoma) is an indolent B-cell lymphoma thought to originate from marginal zone B cells in MALT usually acquired by chronic antigenic stimulation.^1–3^ Initially described by Isaacson and Wright in the stomach with features reminiscent of the Payer’s patch,^4^ cases of MALT lymphoma have been described in virtually any organ, with the most commonly affected sites being the stomach (30−40%) and the ocular adnexa (24%) followed by the parotid and lungs with 11% each, respectively.^1,2^ With the exception of the stomach, the incidence of MALT lymphoma appears to be increasing in most organs. With an incidence of 5–8% of lymphomas diagnosed in adult populations, it is among the more common lymphoma subtypes; it is more common in Asia and constitutes the most commonly diagnosed indolent B-cell lymphoma in China and Korea.

The intriguing occurrence of this type of lymphoma almost exclusively in acquired mucosa-associated lymphoid tissue rather than in native lymphoid structures such as the small intestinal Peyer’s patches has led to the discovery of chronic antigenic stimulation as the underlying cause of MALT lymphoma development.^3,5^ Due to the almost exclusive colonization of the stomach by *Helicobacter pylori*, its role as a causative agent was not only defined for gastric cancer, but early epidemiologic studies have hinted an association with the development of also gastric MALT lymphoma.^6,7^ This was later verified by various *in vitro* experiments as well as by *in vivo* data,^5,8–10^ ultimately resulting in the pivotal paper by Wotherspoon *et al.* and coworkers demonstrating complete regression of gastric MALT lymphoma following sole antibiotic eradication of *H. pylori*.^11^ While this is now recognized as standard therapy for patients with gastric MALT lymphoma irrespective of stage,^12^ identifying other infectious causes has not yielded comparably convincing results although there appears to be some association between *Chlamydophila psittaci* and ocular adnexal lymphomas, at least in certain geographical areas of the world.^3,13–15^ Apart from infections, autoimmune diseases including chronic autoimmune thyroiditis Hashimoto, Sjögren’s syndrome and rheumatoid arthritis have been shown to be associated with MALT lymphomas of various sites.^3^ A retrospective analysis of 158 patients with MALT lymphomas has shown the presence of an autoimmune condition in almost 40%, without impairment of prognosis of such patients in spite of the persistence of the underlying condition after successful therapy of the lymphoma.^16^

Early molecular assessment of apparently lymphoma-free mucosa by histopathology has shown the presence of clonal lymphocytes in patients with gastric MALT lymphoma disseminating within the stomach.^17^ In addition, rather than spreading to lymph nodes and bone marrow, MALT lymphoma has been reported to possess a propensity for homing to other mucosal sites in a relevant proportion of patients. Initially described for gastric MALT lymphoma and the bowel by interaction between alpha4-beta7-integrins and high endothelial venules,^18^ clinical studies have consecutively shown that about 25% of gastric and 40–50% of non-gastric MALT lymphomas present with multifocal organ dissemination.^19,20^ Assessment of monoclonality, however, is not routinely encouraged in patients with suspected or overt MALT lymphoma, as it has also been reported in individuals with *H. pylori* gastritis or after successful treatment of lymphoma.^21^ This state of monoclonality can persist for many years, without being predictive of lymphoma development. According to the present models, the transition from physiological mucosa-associated lymphoid tissue developing in response to (relatively frequent) conditions such as *H. pylori* infection to overt the relatively infrequent MALT lymphoma requires the acquisition of additional genetic anomalies.^5,22^

Data from the 1990s and early 2000s have suggested t(11;18)(q21;q21) as a potential MALT lymphoma-specific event with the potential for prognostic and predictive values.^23,24^ To date, however, varying opinions on the clinical value can be found, and current guidelines are suspiciously vague, as in general there is no predominant pathognomonic hallmark of genetic changes in MALT lymphoma, but the lymphoma at different sites display variable genetic features.^3,22,25^

The objective of this article therefore is to briefly sum up the current knowledge on genetic events that have potential clinical application in patients with MALT lymphomas. For more broad summary of genetic changes and their oncogenic molecular mechanisms on MALT lymphoma, please refer to other reviews.^22,26,27^

## Pathogenesis of MALT lymphoma and molecular events

**Figure 1.**
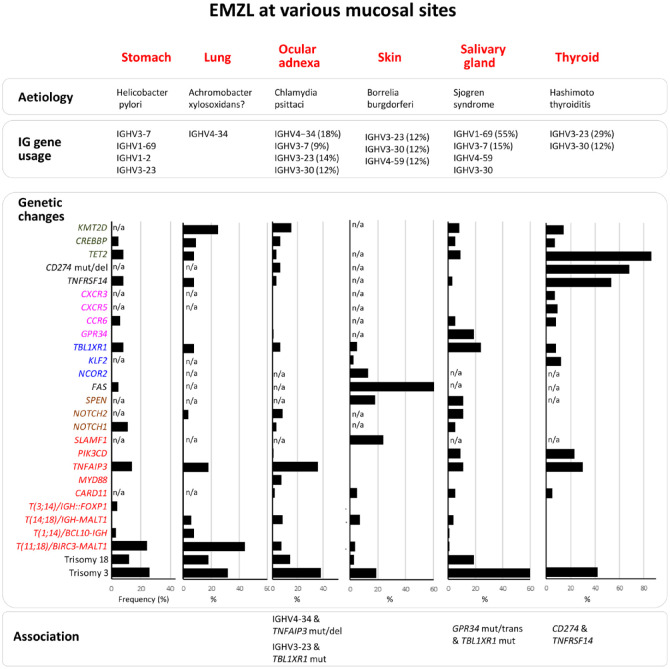
Etiology, immunoglobulin gene usage, recurrent genetic abnormalities in EMZL of various sites. Many of the EMZL-derived immunoglobulins are autoreactive, and where possible the estimated frequency of their usage is provided.^42–47^ As many of the genes involved in EMZL have not been uniformly investigated across different sites, only the recurrent genetic changes are presented in the figure^25,26,43,48–59^. The genes are grouped in different color scheme according to their function. Known association among immunoglobulin usage and genetic changes is indicated. n/a, data not available. Mut, mutation; del, deletion.

As MALT lymphoma commonly originates from a background of a prolonged inflammatory disorder, the evolution of the neoplastic B-cell clone and its clonal expansion are critically influenced by the immunological responses resulting from the inflammatory processes (Figure 1). The development of gastric MALT lymphoma is causatively linked to chronic *H. pylori* infection by epidemiological, in vivo and clinical studies, particularly the demonstration of complete lymphoma remission following *H. pylori* eradication.^6,7,11,12,28,29^ A similar pathogenic role has been suggested for *Campylobacter jejuni* infection in immunoproliferative small intestine disease (alpha heavy chain disease),^30,31^*Borrelia burgdorferi* and *C. psittaci* infection in cutaneous and ocular adnexal MALT lymphoma, respectively,^32–37^ although these microbial associations vary remarkably in different geographical regions. Apart from these microbial etiologies, the MALT lymphomas of the salivary gland and thyroid are almost invariably arising from a background of Sjögren syndrome and Hashimoto thyroiditis, respectively.^38–41^

More recently, IgG4-related disease has been included in the most recent WHO classification under the tumor-like lesions with B-cell predominance^2^ and has also been attributed to chronic antigenic stimulation. In view of this, some authors have reported a potential relationship of IgG4-positive lymphoma, mostly in the ocular adnexa/lacrimal gland to MALT lymphoma. While prognosis appears to be unrelated to IgG4 expression, the pathogenesis and underlying physiological/molecular events might be different for IgG-positive versus IgG4-negative lymphomas, although the clinical relevance of these findings is yet unclear.^60–62^

Although not fully investigated, multiple immunological stimulations resulting from the inflammatory processes are involved in the pathogenesis of MALT lymphoma. B-cell receptor (BCR) signaling is likely relentlessly activated by antigens, most likely autoantigens, as evidenced by biased usages of immunoglobulin genes and expression of stereotypic BCR in MALT lymphoma.^42–47^ In gastric MALT lymphoma, *H. pylori*-mediated T-cell help is known to provide growth stimulation to malignant B cells, involving both cognate interactions and stimulation by soluble ligands such as CD40L and BAFF.^9,63–66^ In addition, innate immune responses are involved in the pathogenesis of MALT lymphoma through activation of the surface receptors GPR34 and CCR6 by their ligands, which are generated by lymphoepithelial lesions or inflamed epithelium.^67,68^

Apart from the immunological drive, the inflammatory processes also promote the acquisition of genetic changes.^22^ The occurrence of the genetic changes is critically influenced by the distinct etiologies associated with MALT lymphoma of various sites, most likely ‘nurturing’ or selecting those bearing cooperative oncogenic activities with the immune receptor signaling as mentioned above. Apart from trisomy of chromosomes 3 and 18 that are common to MALT lymphomas of all sites,^48–50^ all other genetic changes identified so far vary considerably among different sites, but nonetheless affect common molecular pathways important for marginal B-cell biology (Figure 1).

t(11;18)(q21;q21)/BIRC3::MALT1 occurs frequently in MALT lymphoma of the stomach (24%) and lung (40%), but rarely in other sites.^25,51^ t(1;14)(p22;q32)/IGH::BCL10, t(14;18)(q32;q21)/IGH::MALT1 and t(3;14)(p14;q32)/IGH::FOXP1 are infrequent, and lead to overexpression of the involved oncogene, respectively.^52,53,69^ BCL10 and MALT1 are a component of the signaling complex connecting BCR to the canonical NF-kB pathway, and their overexpression by translocation causes constitutive NF-kB activities. The BIRC3::MALT1 chimeric fusion acquires novel functional properties, capable of activating both canonical and non-canonical NF-kB pathways.^27,70^ FOXP1 is a transcription factor, represses the transcription of proapoptotic genes and coordinates NF-kB activities to promote cell survival.^71^

Apart from the above translocations, NF-kB pathway is also affected by inactivation of TNFAIP3 (formerly known as A20) by mutation and/or deletion, which is a global negative regulator of the canonical NF-kB pathway. TNFAIP3 mutation/deletion occurs more frequent in ocular adnexal MALT lymphoma, and are often associated with IGHV4-34 BCR, suggesting their potential cooperation in maintaining NF-kB activation.^54–56^

Several G-protein-coupled receptors (GPCR) are affected by activating genetic changes in MALT lymphoma. GPR34 mutation and rarely t(X;14)(p11;q32)/IGH::GPR34 occur exclusively in MALT lymphoma of the salivary gland (16%), while CCR6 mutations are seen at low frequencies in those of the salivary glands, stomach and thyroid.^43,57^ These genetic changes enhance their receptor signaling, particularly in the presence of their ligands, which are generated by lymhoepithelial lesions or inflamed epithelium.^67,68^

In contrast, thyroid MALT lymphomas show highly frequent and concurrent inactivating mutations in TET2, CD274 and TNFRSF14.^48,57^ CD274 (PD-L1*)* and TNFRSF14 are ligands for co-inhibitory receptor PD1 and BTLA on T-helper cells, respectively, and their inactivation may indirectly promote T-cell help to malignant B cells, thus contributing to lymphomagenesis.^57^

Other recurrent genetic changes are summarized in Figure 1, but not discussed in detail due to lack of clinical relevance.

## t(11;18)(q21;q21) and its clinical implications

MALT lymphoma, as already highlighted above, is not universally characterized by any specific genetic aberration as opposed to other low-grade B-cell lymphoma such as t(14;18)(q32;q21) in 85% of follicular lymphomas, and t(11;14)(q13;q32) in 95% of mantle cell lymphomas.^2^ Among the genetic changes seen in MALT lymphoma, t(11;18)(q21;q21)/*BIRC3::MALT1* is specific to MALT lymphoma, but mainly seen in those of the stomach and lung.^22,25,27^ In these circumstances, detection of this translocation may support MALT lymphoma diagnosis, although most diagnosis could be made based on morphology and immunophenotype.

## Staging and dissemination

Due to the mucosa-specific homing properties of MALT lymphomas, the value of standardized staging was prospectively studied in a large series of 140 patients including 61 gastric and 79 extragastric MALT lymphomas.^19^ A higher than expected rate of multiorgan involvement was seen at 25% of gastric and 46% of non-gastric MALT lymphomas in this study, and sufficient material for genetic analysis had been available in 106 patients, including 44 cases of gastric MALT lymphoma. In total, 19/44 gastric and 6/8 pulmonary MALT lymphomas were positive for t(11;18)(q21;q21), and the translocation was significantly associated with disseminated disease in gastric MALT lymphoma. In patients with non-gastric MALT-lymphoma, dissemination was significantly more common in patients with trisomy 18.

While these data suggest more widespread disease in patients with gastric lymphoma and t(11;18)(q21;q21) as well as trisomy 18 in non-gastric MALT lymphomas, no influence on therapeutic outcome and survival, however, could be documented in these 140 patients.^19^

In a large series from Japan, t(11;18)(q21;q21) was detected in 18 of 87 gastric MALT lymphomas (21%), but was not associated with more disseminated disease in this retrospective analysis. Again, however, no difference in event-free survival and overall-survival at 5 years was found in this study.^72^

## Therapeutic implications

### *H. pylori* eradication/antibiotic therapy

According to the current literature, t(11;18)(q21;q21) is the most widely studied genetic aberration in terms of prognostic and predictive value in patients undergoing antibiotic therapy. Gastric MALT lymphoma has been studied most extensively due to the relatively high rate of t(11;18)(q21;q21) as well as the clearly established role of antibiotics for first-line therapy of gastric MALT lymphoma.^3^

Following the initial reports on the success of *H. pylori* eradication,^73–75^ larger series have been investigated to identify optimal candidates for sole antibiotic therapy as it was noted that a percentage of patients would not respond to antibiotics. Subsequently, various factors including depth of invasion as assessed by endosonography, negativity for *H. pylori* and the presence of t(11;18)(q21;q21) translocation were defined as adverse prognostic factors for response to antibiotic therapy. An analysis of 111 patients with *H. pylori*-positive gastric MALT lymphoma undergoing eradication^76^ showed t(11;18)(q21;q21) in only 2 of 48 complete responders, both of whom relapsed later on, while it was found in 42 of 63 nonresponsive cases. In addition, clinical factors such as the presence of an underlying autoimmune disease^16,77^ and the nuclear expression of BCL10 or NF-kB were demonstrated to be significantly associated with independence of the lymphoma growth from *H. pylori*-mediated stimulation both in individuals with and without the t(11;18)(q21,q21) translocation.^78–80^

The largest retrospective series including 420 patients from Japan confirmed a response rate of 77% to *H. pylori* eradication with subsequent relapse in 10 patients (3.1%) within an observation period of ranging between 3 and 14.6 years.^81^ In this study, non-superficial endoscopic appearance was found to be an adverse prognostic factor, and the already known risk factors including negativity for *H. pylori*, depth of invasion as well as t(11;18)(q21;q21). The latter was found in 30/206 patients analyzed, with 3/30 translocation positive lymphoma responded to antibiotics as opposed to 130/176 translocation-negative cases.

While these data demonstrate a high incidence of t(11;18)(q21;q21) and nuclear BCL10/NF-kB expression in patients whose lymphomas do not respond to antibiotic therapy, responses may occur in patients tested positive for the translocation. Thus, the current guidelines do not recommend routine molecular testing before initiation of *H. pylori* eradication for gastric MALT lymphoma, which is the standard of care irrespective of staging.^12,29^

In addition to gastric MALT lymphoma, a single case showed regression of MALT lymphoma of the bladder following antibiotic therapy in spite of the presence of t(11;18)(q21;q21).^82^

While the rate of *H. pylori* infection in gastric MALT lymphoma has been judged to be in the range of up to 90% in initial reports, the percentage of apparently *H. pylori*-negative cases has increased and has been reported to be up to 30% in more recent series.^83–85^

Early studies have suggested a high rate of genetic aberrations in patients with *H. pylori*-negative gastric MALT lymphomas, with t(11;18)(q21;q21) reported in more than 50% of this subgroup of patients in early studies.^86^ In this series, results again suggested a correlation of t(11;18)(q21;q21) with more advanced stages.

Somatic mutations and their influence on long-term outcome were evaluated in a series of 57 *H. pylori-*negative gastric MALT lymphoma patients, with targeted sequencing data available in 35/57 patients (lymphoma-specific 93-gene panel).^87^ As expected, alterations in NF-κB signaling were the most frequent finding. In total, 40% harbored a somatic mutation affecting the NF-κB pathway (e.g. TNFAIP3 = 23%, CARD11 = 9%, MAP3K14 = 9%); in addition, a *MALT1* rearrangement was detected in 39% (22/57) of cases. Thus, in summary 86% of assessed cases were affected by NF-κB pathway alterations. Further somatic mutations detected included frequently *NOTCH1* and *NOCTH2, KMT2D* and *CREBPP*. With regard to clinical impact, no specific somatic changes or alterations in the NF-κB signaling pathway had a significant impact on the progression-free survival following various treatment approaches but there was a nonsignificant tendency toward worse outcome in patients presenting with *KMTD2* mutation (*p* = 0.125) or *MAP3K14* mutation (*p* = 0.18). While again no conclusion for clinical application can be drawn from these data, the results confirmed the strong impact of the NF-κB pathway also for *H. pylori* independent MALT lymphoma.

## Systemic treatment

As has been discussed above, the presence of t(11;18)(q21;q21) appears to increase the likelihood of non-response to *H. pylori* eradication, but does not confer total resistance to antibiotic therapy. As this might be mostly due to inhibition of apoptosis due to various potential mechanisms,^72^ it has been hypothesized that this might also result in decreased response to various systemic therapies applied in patients with gastric lymphoma not responding to eradication or in patients positive for t(11;18)(q21;q21) with disseminated disease from other MALT lymphoma sites. In view of this, the influence of t(11;18)(q21;q21) has been studied for various treatments in patients either failing HP eradication for gastric MALT lymphoma or being treated upfront.

Various caveats, however, have to be kept in mind when interpreting these reports. First of all, the definition of ‘non response’ has not always been uniformly applied in those (mostly small, and partly retrospective) series. In fact, a large majority of patients with gastric MALT lymphoma would probably not have been treated according to modern guidelines, as they had either persisting, but stable lymphoma or residual disease after partial regression following HP eradication. Current guidelines^12,29^ suggest that most of these patients do not require further therapy based on two large published studies. In the first trial, patients following eradication of *H. pylori* were randomized to either oral chlorambucil or watch-and-wait.^88^ Out of 110 patients randomized, the large majority were in CR (63/110), 23 were rated as being in PR following *H. pylori* eradication, 15 had stable disease while 9 had unknown disease status. No significant difference, however, was seen between the treatment arms in terms of progression-free survival at 5 years (21% in the observational arm *versus* 11% for chlorambucil) as well as for overall survival. No data, on t(11;18)(q21;q21) in the study population, were reported. An observational study by Fischbach *et al.* and coworkers including 108 patients with minimal residuals at 12 months following eradication therapy showed that 32% developed CR with prolonged follow-up, 62% remained stable while only 6% progressed after a median follow-up of 42 months.^74^

In terms of systemic treatment for disseminated or symptomatic MALT lymphoma, several series have evaluated the impact of t(11;18)(q21;q21) on response to treatment and long-term results. Again, this has to be interpreted with caution as treatment had not been uniformly defined and varied between studies.

According to current guidelines,^12^ combinations of chlorambucil ± anti-CD20-antibody rituximab (R) – based on the only randomized phase III trial conducted in this indication – and R-bendamustine, in analogy to other indolent B-cell lymphomas, are the most commonly applied regimens in this setting.

The three-armed IELSG-19 trial allocated 454 untreated MALT lymphoma patients with both gastric and extragastric origin to chlorambucil monotherapy *versus* R-chlorambucil *versus* R-monotherapy, and confirmed a benefit for the addition of R to chlorambucil regarding the primary endpoint 5-year event-free survival (EFS) (51% *versus* 68%, *p* = 0.009).^89^ Despite a comparable outcome for both monotherapy arms and no difference at all in overall survival, this study has set a potential treatment standard for many centers. No data on t(11;18)(q21;q21) or further predictive clinicopathological markers were evaluated in this study. In a study on the activity of R monotherapy in patients with gastric MALT lymphoma, however, no influence of t(11;18)(q21;q21) was reported by Martinelli *et al.* and coworkers.^90^ Analysis for t(11;18)(q21;q21) could be performed in 21/26 evaluable patients and detected the translocation in 8 (38%) of these patients, who had all relapsed from *H. pylori* eradication ± systemic therapy or were unsuitable for antibiotic therapy alone. Clinical response was reported in 75% of patients with and 69% of patients without t(11;18)(q21;q21).

As opposed to this, in an early retrospective series failure to oral alkylating agents was reported in 7/12 patients with t(11;18)(q21;q21)-positive gastric MALT lymphoma, while only one treatment failure was reported in nine patients without the respective translocation.^91^ Furthermore, the remission was durable in 8/9 patients without t(11;18)(q21;q21) *versus* only in one-twelfth patients in the translocation-positive cohort. In the following, the same French group published a series of 13 patients with t(11;18)(q21;q21)-positive gastric MALT lymphoma treated with chlorambucil plus R, and suggested a higher sensitivity of these patients to combination treatment as CR was achieved in all patients.^92^

In analogy to patients following *H. pylori* eradication, t(11;18)(q21;q21) was still detectable by real-time PCR during long-term follow-up in 70% of cases, despite continuous macroscopic and histological remission.^21^ In addition, persistence of B-cell monoclonality was seen in 30% of these patients. Clinical value, however, appears negligible, as both findings are apparently not associated with clinical relapse, which had also been shown for patients in remission following various systemic approaches, but also eradication of *H. pylori*.^21,93^

To further confirm the impact of translocations status on the combination of R-chlorambucil and R monotherapy, respectively, a larger observational study in t(11;18)-positive (*n* = 31) *versus* negative (*n* = 18) gastric MALT lymphoma patients was conducted again by the same investigators.^94^ In line with their previous results but not the data reported by Martinelli *et al.* and coworkers in 26 patients,^90^ the combination was significantly more effective than R-monotherapy in the t(11;18)(q21;q21)-positive cohort (objective response in 92% *versus* 55%, *p* = 0.07), while R-monotherapy was equally effective in t(11;18)(q21;q21)-negative patients (objective response in 83% *versus* 92%, *p* = 0.47).^94^ In contrast to these data, a recent follow-up publication suggested a superior 5-year PFS for the combination also in a t(11;18)-negative cohort of 71 patients (54 treated with R alone and 17 with the combination; 5-year PFS 60% for R and 88% for R-chlorambucil, *p* = 0.05).^95^

Most data regarding R-bendamustine derive from follicular lymphoma but a Spanish phase II trial assessed a response-dependent protocol of 4–6 cycles R-bendamustine in 60 MALT lymphoma patients.^96,97^ The overall response rate was 100% (95% CI 93-100) and the EFS at 7 years remarkably high at 87.7%.^96,97^ In this trial, t(11;18)(q21;q21) was detected in 9 patients (2 gastric, 3 lung, 2 multi focal, 1 colorectal, 1 skin) and outcome was equal to patients with no translocation in view of numbers of required cycles, EFS, PFS, and OS.

In addition, mostly smaller reports have also assessed the impact of t(11;18)(q21;q21) in less commonly applied systemic therapies. Translocation t(11;18)q21;q21) was shown not to be predictive of response to treatment with cladribine in 17 gastric MALT lymphoma patients. In a Chinese series of 10 patients, the authors reported increased efficacy of salvage thalidomide in patients with t(11;18)-positive MALT lymphoma.^98,99^

In summary, while some groups adapt their treatment based on these data and prefer particularly chlorambucil-combination therapy for t(11;18)(q21;q21) cases,^100^ given the retrospective nature, limited number of cases, missing impact on survival and not at least also somewhat contradictive data, no definite conclusion regarding the impact of t(11;18)(q21;q21)-translocation status and chemotherapy-based systemic therapy could be drawn.

## Novel (targeted) therapies

### Immunomodulatory imide drugs – lenalidomide

In view of the overlap in molecular mechanism, that is, NF-κB pathway activation by genetic changes, between MALT lymphoma and multiple myeloma, the immunomodulatory imide drug (IMiD) lenalidomide (LEN) has been tested for treatment of MALT lymphoma.^101^ Two phase II studies with a distinct cohort of MALT lymphomas (one with LEN monotherapy and the other with the combination of *R* + LEN) have shown promising response rates of ORR 61% (CR rate: 33%) for monotherapy and 80% ORR (CR rate: 54%) for R-LEN.^102,103^ As MUM1 and cereblon have been shown to be markers for response to LEN-based therapies in myeloma, 46 patients out of these two trials were retrospectively tested for expression of these two potential markers using immunohistochemistry.^104^ In total, 54% stained positive for cereblon and 38% for MUM-1, but no significant difference in terms of response could be found between cereblon-positive *versus* -negative patients (68% *versus* 86%, *p* = 0.16) or MUM-1 expressors (RR 71% *versus* 79%, *p* = 0.55), with no difference for PFS.

## Bruton tyrosine kinase inhibitor

More recently, also trials using Bruton tyrosine kinase (BTK) inhibitors in patients with marginal zone lymphomas including MALT lymphoma have been performed.^105^ In an initial series using ibrutinib, 32/63 patients included suffered from MALT lymphoma, and in the overall cohort a response rate of 53% with an 18 months PFS of 62% was seen.^106^ An update published in 2020 showed an RR of 65% in the MALT lymphoma cohort.^107^ In this series, also biomarker testing was performed and available for 24 patients with MALT lymphoma. In this series, patients with *TNFAIP3* (*A20*) mutations (*n* = 10) had significantly greater shrinkages of their lymphomas than patients with wild type (*p* = 0.0386).

In addition, variants in *KMT2D* (8/15 patients tested) as well as *CARD11* (5/25 patients tested) had a worse in outcome in terms of duration of response for the further and PFS for CARD11 when patients with MALT lymphoma were analyzed.

The MAGNOLIA trial has tested the application of zanubrutinib in patients with marginal zone lymphoma, including 26 patients with MALT lymphoma (out of a total of 68 patients).^108^ An overall RR of 68% was obtained, and as the results of this trial, zanubrutinib was approved for treatment of all subtypes of marginal zone lymphoma after prior therapy with an anti-CD20 antibody. A biomarker analysis was carried out in 17 patients,^109^ including 8 with MALT lymphoma, by using whole exome sequencing. In the total cohort, *MYD88* and again *TNFAIP3* mutations correlated with a prolonged PFS, while *KMT2D* mutations had a PFS of 13.4 *versus* not reached in the overall cohort. While the number of patients is small and especially the subgroup of patients with MALT lymphomas is limited, these data are in line with the results reported for ibrutinib in terms of potential impact of *TNFAIP3* and *KMT2D* mutations. Nonetheless, this requires further study in the future.

## Conclusion

As opposed to other B-cell lymphomas, MALT lymphomas are genetically heterogeneous and show varying genetic changes according to localization. While especially t(11;18)(q21;q21) is associated with more widespread disease and potential resistance to *H. pylori* eradication in gastric MALT lymphoma, these results do not necessitate routine molecular assessments before initiation of therapy with antibiotics, as translocation-positive lymphoma might respond *to H. pylori* eradication. In view of this, the application of alternative therapies in patients with gastric MALT lymphoma harboring t(11;18)(q21;q21) as first-line management is not justified. In addition, currently there are no firm data showing an influence of genetic aberrations on other types of therapy including R monotherapy and chemotherapy with two CdA. While retrospective analyses have produced conflicting data in terms of response to R plus chlorambucil, large prospective series are missing. Two molecular analyses of small subgroups of patients treated with the BTK inhibitors ibrutinib and zanubrutinib have both suggested that mutations in *TNFAIP3* (*A20*), *KMT2D* and probably *CARD11* might influence response to therapy. However, in the absence of larger prospective data, management of patients should not be based on individual analysis of genetic changes for the time being.

## Declarations

**Ethics approval and consent to participate:** Not applicable.

**Consent for publication:** Not applicable.

**Availability of data and materials:** Not applicable.
